# School completion and progression to higher education in adolescents with social anxiety: a linkage between Young-HUNT3 and national educational data (2008–2019), Norway

**DOI:** 10.1186/s12889-024-18271-w

**Published:** 2024-03-18

**Authors:** Ingunn Jystad, Tommy Haugan, Ottar Bjerkeset, Erik R. Sund, Tore Aune, Hans M. Nordahl, Jonas R. Vaag

**Affiliations:** 1 Faculty of Nursing and Health Science, Nord University, 7601 Levanger, Postbox 93, Norway; 2 Department of Public Health and Nursing, Faculty of Medicine and Health Sciences, Norwegian University of Science and Technology, Trondheim, Norway; 3 Levanger Hospital, Nord Trøndelag Hospital Trust, Levanger, Norway; 4 The Norwegian Directorate for Children, Youth, and Family Affairs, Bufetat, Norway; 5 Department of Mental Health, Norwegian University of Science and Technology, Trondheim, Norway; 6 Department of Psychology, Inland Norway University of Applied Sciences, Lillehammer, Norway

**Keywords:** Adolescents, Education, Mental Health

## Abstract

**Background:**

Social anxiety disorder (SAD) most commonly develops in adolescence—a period of life that includes a transition to upper secondary school. The aim of this study is to investigate the extent to which social anxiety in adolescence is associated with the completion of upper secondary school and progression to higher education.

**Methods:**

This longitudinal study includes 8,192 adolescents aged 13–19 years who participated in the Norwegian Young-HUNT 3 population-based study. Social anxiety is measured employing (1) diagnostic interview screening questions (interview) and (2) a self-reported symptom index (questionnaire). Notably, we define the cohorts based on these two methods. Using national educational data (2008–2019), we follow educational attainment among the cohorts until they turn 25 years of age.

**Results:**

We found that adolescents who screened positive (SP) for SAD had a predicted probability of upper secondary school completion at 21 years of age that was 14% points lower than those who screened negative (SN). Further, differences remained when looking at completion rates at age 25 years. Moreover, predicted probabilities for completion were inversely associated with increasing levels of self-reported social anxiety symptoms. Similarly, the proportion of the completers of an academic program in the SP group that were enrolled in higher education by 25 years of age, were lower than for the SN group (87 vs. 92%).

**Conclusion:**

Social anxiety in adolescence, both self-reported symptoms and diagnostic screening, has long-term negative impact on upper secondary school completion and to some extent enrollment to higher education.

**Supplementary Information:**

The online version contains supplementary material available at 10.1186/s12889-024-18271-w.

## Background

Social anxiety disorder (SAD) and social anxiety symptoms most often emerge during adolescent years [1, 2] —a phase of life during which educational choices and performance provide an important foundation for future health and socioeconomic prospects [3–5]. The completion of upper secondary school (equivalent to senior high school) provides academic and/or vocational qualifications that lead to a higher likelihood of successful participation in the labor market [6]. Non-completion of upper secondary school, on the other hand, is not only related to adult unemployment and low income [6], but also to poor health, as well as an increased risk of a need for disability pension [5, 7–10].

SAD and social anxiety at subclinical levels include symptoms of excessive fear and avoidance of social and performance situations [11]. Therefore, school may represent one of the most anxiety-provoking settings for adolescents with social anxiety [12–14]. Compared to class mates, pupils with SAD are more likely to report school-related impairment during adolescence [15]. Often, they experience that their social fears interfere with school activities [16]. In a Finish study of 784 adolescents aged 12–17 years, pupils with SAD or subclinical SAD reported lower grades compared to their healthy class mates [17]. There is also some indication for lower rates of upper secondary school completion among individuals with SAD [18–20]. Data from the Mental Health Supplement to the Ontario Health Survey of more than 8000 Canadian adults reported that a lifetime diagnosis of SAD is associated with a higher risk of dropping out of high school [18]. Furthermore, a longitudinal study of 15,755 Swedish individuals with SAD demonstrated underachievement in all levels of education, with the greatest impairment in upper secondary school completion [20]. In a US study of 201 adults with anxiety disorders, where 26% had not completed high school, the majority described typical SAD symptoms as their main reason for leaving [19]. Individuals with elevated social anxiety are also less likely to have aspirations for higher education [21], and there is evidence that diagnosed individuals are less likely to begin and complete a university degree [20, 22].

However, previous research on social anxiety and educational attainment has mostly used cross-sectional designs and/or retrospective data collection [18, 19], the latter involving a risk for recall bias [23]. Furthermore, several studies have investigated social anxiety as part of internalized disorders or anxiety disorders in general [24–26], while others have been based on diagnosed individuals [19, 20]. Since people with SAD tend not to seek professional help for their problems [27], diagnosed individuals might represent a highly selective group and, potentially, those with the most severe forms of SAD. There is evidence, though, that individuals with subclinical symptom levels of SAD also experience great suffering and loss of function [28–31]. Therefore, the use of various measures of social anxiety on a non-selective, population-based sample is advantageous. To the best of our knowledge, there are no prospective, longitudinal, population-based studies among adolescents with social anxiety investigating registry based educational attainment in relation to both self-reported symptom severity (questionnaire) and their diagnostic screening status (interview).

We use data from a large Norwegian population-based study (Young-HUNT 3) linked to registry data from The Norwegian National Education Database (NUDB) to investigate the associations between social anxiety, measured through both screening outcomes for SAD (interview) and self-reported symptom levels (questionnaire), and the completion of upper secondary school by the age of 21 and 25 years, respectively. We also aim to investigate associations between social anxiety and enrollment in higher education.

## Materials and methods

### Study population: participants in the Young-HUNT 3 study (2006–2008)

The Young-HUNT 3 was a cross-sectional population-based study in which all adolescents aged 13–19 years (*n* = 10,464) living in the Nord Trøndelag county of Norway were invited to participate. The study involved a comprehensive self-report questionnaire that 8,199 adolescents completed during school hours (response rate 78.4%), as well as clinical interviews and physical examinations performed approximately one month thereafter. The self-report questionnaire included a broad range of sociodemographic, school, and health-related topics, in addition to a self-report measure of social anxiety symptoms [32]. Further details regarding the HUNT and Young-HUNT are described elsewhere [32, 33]. In addition, the Young-HUNT 3 included a sub study on social anxiety, in which a total of *n* = 6,610 participated in the initial SAD screening phase [34]. Thus, the Young-HUNT 3 included information on both self-reported symptoms and SAD diagnostic screening status.

### Norwegian National Education Database (NUDB)

The NUDB contains individually registered information on educational level and registered students (enrollments) per October 1st, coded according to the Norwegian Standard Classification of Education (NUS) (and corresponding codes to the international education standard [ISCED]) for all Norwegian citizens [35, 36]. The Young-HUNT 3 dataset was merged through a unique citizen identifier with NUDB for the period of 2008–2019, enabling a follow-up period for each participant until the age of 25 years. However, due to a lack of identification codes *(n =* 2) and being born before 1987 *(n =* 5), seven of the participants were excluded, resulting in a total of *n* = 8,192 individuals.

### Variables

#### Educational outcomes

*Completion of upper secondary school.* Although education is mandatory only up to 16 years of age, almost all Norwegian adolescents (98%) continue to upper secondary school [37]. Upper secondary school offers academic programs (also known as general programs) (lasting three years, primarily providing university qualifications), as well as vocational programs combining theory and practical learning (lasting two to four years, primarily providing vocational qualifications). Additionally, vocational programs offer the possibility to take additional courses and gain the qualifications necessary for higher education [38]. In this study, we chose two definitions for the completion of upper secondary school: graduation by 21 and 25 years of age, respectively. The age of 21 years was chosen because graduation within five years has been a demarcation point in previous studies based on NUDB data [7, 39–41]. The age of 25 years was chosen to account for the flexibility of the Norwegian school system, and because this was the age of the youngest participants in the latest year of the register data. For descriptive statistics, we categorized those who had completed (= 1) and not completed (= 0) upper secondary school by the age of 20, 21, 22, 23, 24, and 25 years, respectively. We categorized completion of educational program (academic and vocational) based on a standard classification from Statistics Norway using the participants’ NUS code the year they completed upper secondary school [42].

*Higher education.* We investigated enrollment in higher education by 25 years of age. Thus, 1 = those who were in higher education by the age of 25 years; 0 = those who were not in higher education by the age of 25 years. Since our observations from NUDB data were from 2008 to 2019, the observation time for the oldest adolescents (born in 1987) was from 21 to 25 years of age, while the majority was observed from 19 to 25 years of age.

#### Exposure variables from the Young-HUNT 3

*Social anxiety screening status (n =* 6,609) was obtained by interview, using the following three introductory items from the social anxiety part of the Anxiety Disorders Interview Schedule for Children (ADIS-C) (yes/no answers) [43, 44]: “*When you are with others, at school, in restaurants, or at parties, do you ever feel that people might think that something you do is stupid or dumb?”; “When you are with other people at school, restaurants, or parties, do you think that people might laugh at you?”; “When you are in these situations with others (school, restaurants, and parties), do you worry that you might do something that will make you feel ashamed or embarrassed?”*. The participants who answered “yes” to at least one of these were considered SP for SAD *(n =* 388), while those who answered “no” to all three items were considered SN (*n* = 6,221) [34].

*Self-report information on social anxiety symptoms (the SAD symptom index)* was based on the six items included in the questionnaire assessing symptoms of SAD. The six items were selected for the Young-HUNT from the Social Phobia and Anxiety Inventory for Children (SPAI-C) [45, 46] and the Social Phobia and Anxiety Inventory (SPAI) [47] through the use of an item analysis approach [48] based on *Diagnostic and Statistical Manual 4th Edition* (DSM-IV) criteria [43, 45, 49]. The items included the following: *I feel anxious and do not know what to do in an embarrassing situation; I feel anxious when I am with others and have to do something while they watch me do it (e.g., be in a play, play music, sports); I feel anxious when I have to speak or read aloud in front of a group of people; Before I go someplace where I am going to be with people (e.g., a party, school, football game), I sweat, my heart beats fast, and/or I get a headache or stomachache; Before I go to a party or someplace with other people, I think about what could go wrong (e.g., that I will make mistakes, seem dumb, and/or they will see how frightened I am); I feel anxious and do not know what to do when I am in a new situation.* Each item was rated on a five-point Likert scale as follows: 1 = *never*, 2 = *seldom*, 3 = *sometimes*, 4 = *often*, and 5 = *always*. For each respondent, a SAD symptom index was constructed by adding up the scores (1 to 5) for all six items covering the social anxiety dimension and dividing the total number of items (Cronbach’s alpha = 0.84). Thus, the resulting mean symptom scale score ranged continuously from 1.0 to 5.0. To date, there exists no established clinical cut-off of this scale.

*Age* was measured in years and used as a categorical variable (with 13 as reference category).

*Family financial status* consisted of an item asking whether the participant ranged their family’s financial situation as 1 = equal, 2 = better, or 3 = worse “compared to others”.

### Statistical analyses

Data management and statistical analyses were performed using Stata version 15.1 [50]. The analyses were performed in three steps: First, *percentages, means, and standard deviations for the variables used in the analyses* were calculated for each study group (i.e., SPs and SNs) and stratified by sex. For the completion of upper secondary school (i.e., the outcome variable), the total numbers and corresponding proportions of each study group who completed upper secondary school by the age of 20, 21, 22, 23, 24, or 25 years were calculated. Among the completers, we calculated the numbers and corresponding proportions of academic and vocational programs. Next, the numbers and corresponding percentages who were registered for higher education by the age of 25 years were calculated among completers of academic programs in each study group.

Second, in *multivariable logistic regression analyses*, associations between social anxiety and the completion of upper secondary school by 21 and 25 years of age were calculated for each measure of social anxiety: (1) their ADIS-C screening status and (2) their SAD symptom index. Due to departure from linearity between the SAD symptom index and log odds of the outcome, the SAD symptom index was also included as a squared variable. Similar analyses were performed using enrollment into higher education as outcome variable. The analyses were controlled for participants’ *sex*, *age at Young-HUNT 3 participation*, and subjective perception of their *family’s financial status*.

Third, based on the logistic regression models we calculated predicted probabilities (i.e. adjusted predictions) for school completion for the SPs and SNs by the ages of 21 and 25 years. Predicted probabilities were also calculated for combinations of screening status and sex, by forming an interaction-term in the regression models. Predicted probabilities were also calculated according to SAD symptom index and sex. Identical analyses were performed using enrollment into higher education as outcome variable. The results are presented in tables and graphs with 95% confidence intervals (CIs).

### Missing data

Information on the highest level of education by the ages of 20–25 years was missing for a total of *n* = 10 of the adolescents. Missing values for the predictor variables and covariates are described in Table 1.

**Table 1:** Descriptive statistics (n%) among Anxiety Disorders Interview Schedule for Children screened-positive and screened-negative adolescents

	Screened negatives *n* = 6,221			Screened positives *n* = 388		
	All *n* *= 6,221*	Females *n* *= 3,063*	Males *n* *= 3,158*	All *n* *= 388*	Females *n* *= 267*	Males *n* *= 121*
**Social anxiety disorder symptom index**						
Mean score (SD)	**1.9 (0.7)**	2.0 (0.7)	1.7 (0.6)	**2.8 (0.9)**	2.9 (0.8)	2.6 (0.9)
*Missing* n *(%)*	**242 (3.9)**	79 (2.6)	163 (5.2)	**19 (4.9)**	14 (5.2)	5 (4.1)
**Age**						
Mean age (SD)	**16.0 (1.7)**	16.0 (1.7)	15.9 (1.7)	**16.1 (1.9)**	16.2 (1.8)	16.0 (2.0)
Age group (*n*%)						
13–15 years	**3,176 (51.1)**	1,552 (50.7)	1,624 (51.4)	**195 (50.3)**	125 (46.8)	70 (57.9)
≥ 16 years	**3,045 (48.9)**	1,511 (49.3)	1,534 (48.6)	**193 (49.7)**	142 (53.2)	51 (42.1)
**Family’s financial status (** ***n*** **%)**						
Worse than others’	**497 (8.5)**	263 (8.9)	234 (8.0)	**57 (15.9)**	47 (18.9)	10 (9.2)
Equal to others’	**4,310 (73.4)**	2,223 (75.6)	2,087 (71.3)	**250 (69.8)**	173 (69.5)	77 (70.6)
Better than others’	**1,064 (18.1)**	456 (15.5)	608 (20.8)	**51 (14.3)**	29 (11.6)	22 (20.2)
*Missing*	**350 (5.6)**	121 (4.0)	229 (7.3)	**30 (7.7)**	18 (6.7)	12 (9.9)
**Completion of upper secondary school (** ***n*** **%)**						
≤ 20 years	**4,248 (68.9)**	2,317 (76.2)	1,931 (61.7)	**211 (55.5)**	153 (58.4)	58 (49.2)
≤ 21 years	**4,758 (76.5)**	2,466 (80.6)	2,292 (72.7)	**237 (61.2)**	166 (62.2)	71 (59.2)
≤ 22 years	**4,968 (79.9)**	2,544 (83.1)	2,424 (76.8)	**262 (67.7)**	179 (67.0)	83 (69.2)
≤ 23 years	**5,075 (81.6)**	2,589 (84.6)	2,486 (78.8)	**272 (70.3)**	187 (70.0)	85 (70.8)
≤ 24 years	**5,167 (83.1)**	2,625 (85.8)	2,542 (80.6)	**277 (71.6)**	191 (71.5)	86 (71.7)
≤ 25 years	**5,239 (84.3)**	2,659 (86.9)	2,580 (81.8)	**283 (73.1)**	195 (73.0)	88 (73.3)
*Missing*	**5 (0.1)**	2 (0.1)	3 (0.1)	**1 (0.3)**	0 (0.0)	1 (0.8)
***Educational program***						
Academic program n (%)	**3374 (65.1)**	1984 (75.7)	1390 (54.2)	**165 (59.1)**	118 (61.8)	47 (53.4)
Vocational program n (%)	**1810 (34.9)**	637 (24.3)	1173 (45.8)	**114 (40.9)**	73 (38.2)	41 (46.6)
*Missing* n (%)	55 (1.1)	38 (1.4)	17 (0.7)	4 (1.4)	4 (2.1)	0 (0.0)
**Entered higher education by ≤ 25 years**						
Completers of academic program n (%)	**3092 (91.6)**	1868 (94.2)	1224 (88.1)	**143 (86.7)**	105 (89.0)	38 (80.9)

^*^The percentages of those who completed upper secondary school at ≤ 20 years do not include participants born in 1987 (*n* = 72). Parts of this table are also published in [34], and [21]

## Results

Table 1 shows a descriptive presentation of the two study groups, namely the SP and SN groups. Among the 6,609 adolescents, a total of 388 (5.9%) were SP and 6,221 (94.1%) were SN [34]. From the age of 20 to 25 years, the percentages of the study participants that completed upper secondary school were lower for the SP group at all ages. By the age of 25 years, 84% of the SN adolescents had completed upper secondary school compared to only 73% of the SP adolescents. Furthermore, a total of 92% of the completers of academic programs in the SN group had started higher education by age of 25, compared to 87% of the completers of academic program in the SP group.

### Social anxiety and the completion of upper secondary school and start of higher education

Table 2 presents the predicted probabilities and corresponding 95% CIs for the completion of upper secondary school for each study group (SN and SP), and for enrollment in higher education among completers of academic programs for each study group (SN and SP). SP adolescents had a predicted probability for completion by 21 years of age that was 14% points lower than for the SN group. For completion by 25 years of age, the predicted probabilities were higher for both SP and SN groups, however, still 10% points lower for the SP group. In sensitivity analyses stratifying in two age groups (13–15 and ≥ 16 years at baseline), we observed that the predicted probabilities for completion of upper secondary school by 21 and 25 years were lower for SP adolescents 13–15 years of age (58% and 72%), than those of SP adolescents aged 16 years and older (67% and 77%).

The predicted probability for enrollment in higher education by 25 years was 6% points lower for the completers of academic program in the SP group, than for the completers of academic program in the SN group (86% vs. 92%).

**Fig. 1:**
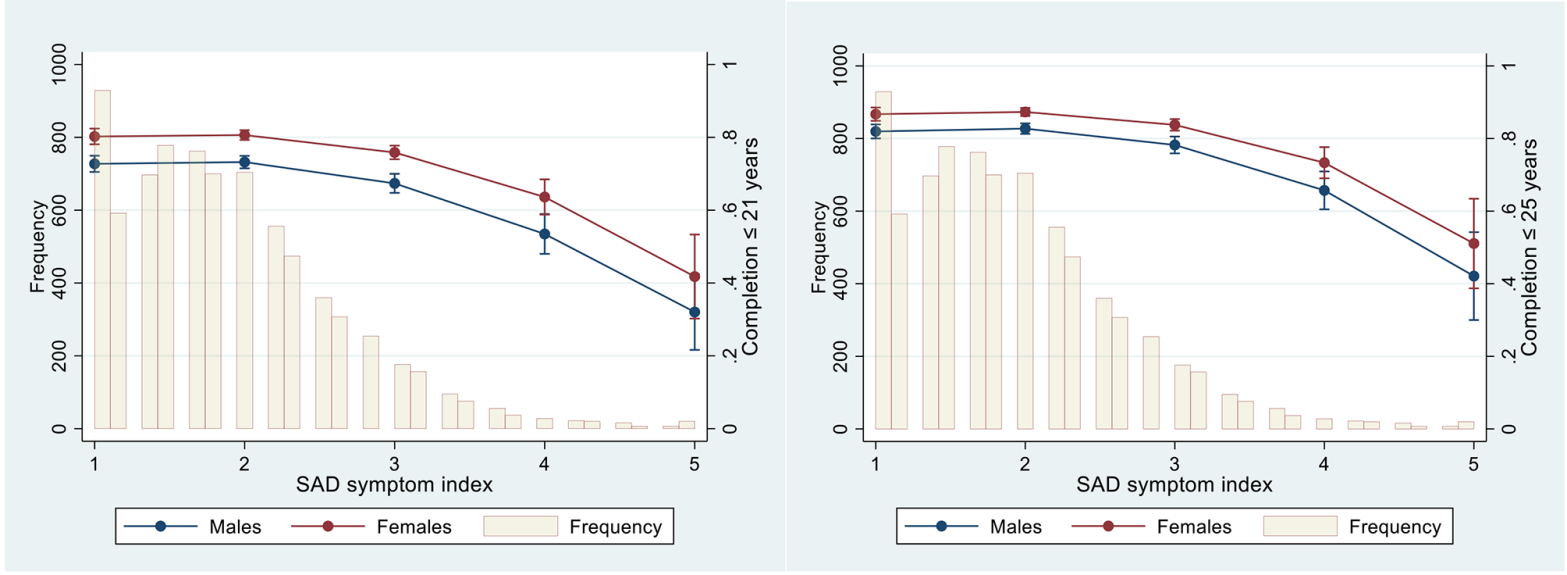
Predicted probability and 95% confidence intervals for the completion of upper secondary school by ≤ 21 and ≤ 25 years of age according to sex and social anxiety disorder symptom index values of 1–5, and frequency of social anxiety disorder symptom index^1^ (bars) Group sizes due to missing data for the predictor, covariate, or outcome variable—Total sample: *n* = 7,451, males: *n* = 3624, females: *n* = 3827 ^1^ The SAD symptom index is a mean score based on six items assessing social anxiety symptoms in various social situations, each rated on the following Likert scale: 1 = *never*, 2 = *seldom*, 3 = *sometimes*, 4 = *often*, and 5 = *always*. Thus, a SAD symptom index score of 1 is equal to “never” for all six items, and a SAD index score of 5 is equal to “always” for all six items. Analyses are adjusted for sex, age, and family's financial status.

Figure 1 shows predicted probabilities for upper secondary school completion by 21 (left figure) and 25 (right figure) years of age according to the SAD symptom index for males and females at baseline. For both completion ages, the predicted probability decreased with increasing levels of SAD symptoms in both sexes. The predicted probabilities among those with SAD symptom index of 1 and 2 were fairly similar, following a slight decrease in predicted probabilities when scores of SAD symptoms increased from 2 to 3. Further, from 3 to 5, the decline is at its steepest. For instance, for females who, on average, answered “seldom” to all six self-reported items for social anxiety (i.e., a SAD symptom index score of 2), the predicted probability of school completion by 21 years of age was 81%, whereas the corresponding predicted probability for those females who, on average, answered “often” (i.e., a SAD symptom score of 4) was 64%. The predicted probabilities for completion by 25 years of age were higher for all levels of the self-report, however, still decreasing by elevating symptom levels. In sensitivity analyses stratifying in two age groups (13–15 and ≥ 16 years), we observed that the associations were fairly similar for both groups for completion at 21 and 25 years of age (see Additional file 1: Figure S1 and S2).

**Fig. 2:**
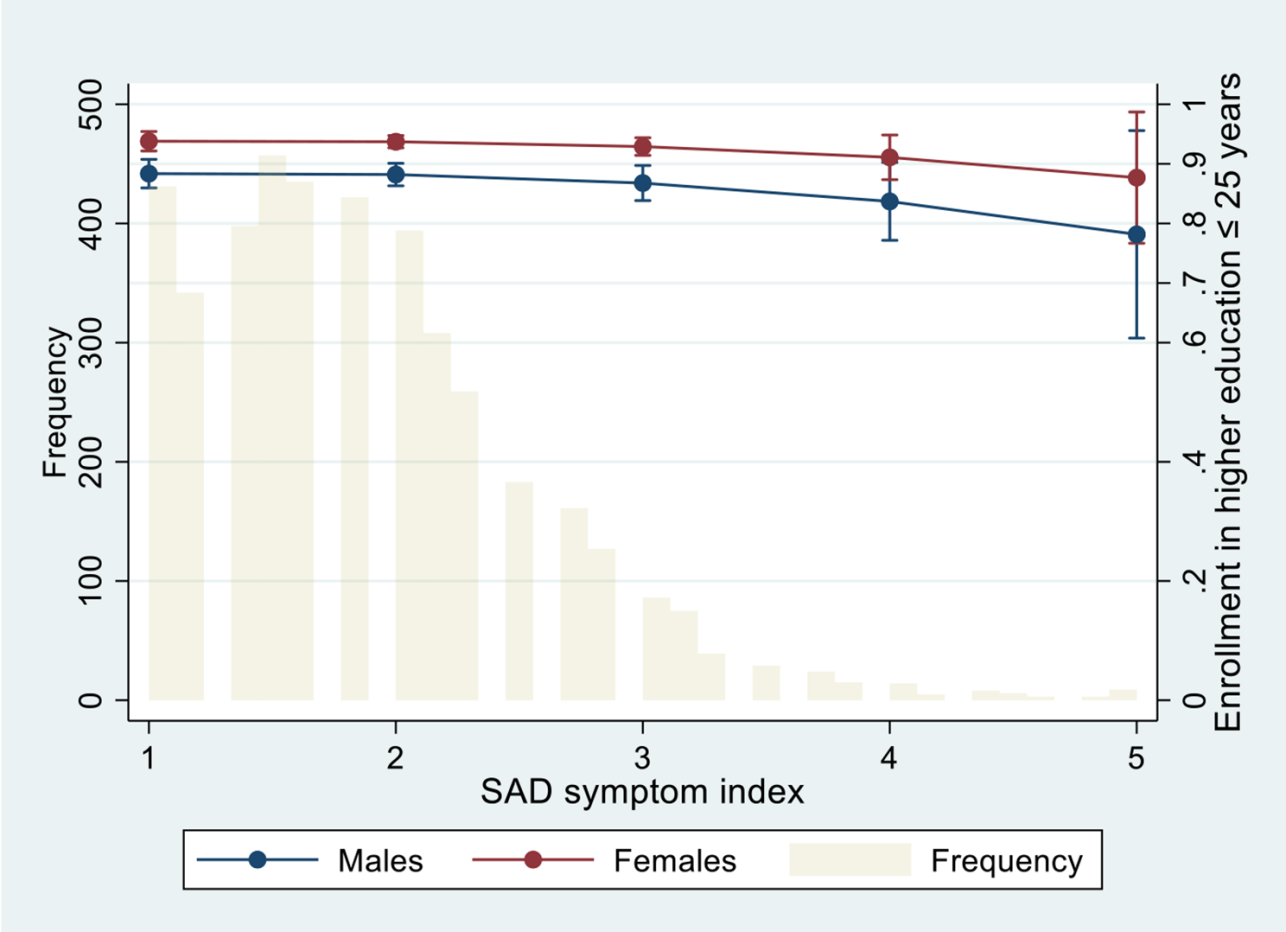
Predicted probability and 95% confidence intervals for enrollment in higher education by ≤ 25 years of age according to sex and social anxiety disorder symptom index values of 1–5, and frequency of social anxiety disorder symptom index^1^ (bars). Group sizes due to missing data for the predictor, covariate, or outcome variable: Total sample: n = 4083, males: n = 1641, females: n = 2442. ^1^The SAD symptom index is a mean score based on six items assessing social anxiety symptoms in various social situations, each rated on the following Likert scale: 1 = *never*, 2 = *seldom*, 3 = *sometimes*, 4 = *often*, and 5 = *always*. Thus, a SAD symptom index score of 1 is equal to “never” for all six items, and a SAD index score of 5 is equal to “always” for all six items. Analyses are adjusted for sex, age, and family's financial status.

Figure 2 shows predicted probabilities for enrollment in higher education by 25 years of age among completers of academic programs, according to the SAD symptom index for males and females at baseline.

**Table 2:** Predicted probabilities^1^ (with 95% confidence intervals) for the completion of upper secondary school by ≤ 21 and ≤ 25 years and enrollment in higher education by ≤ 25 years of age per ADIS-C* screening status and the latter screening status in combination with sex

	Screened negatives *n* = 6,221			Screened positives *n* = 388		
	All *n* *= 6,221*	Females *n* *= 3,063*	Males *n* *= 3,158*	All *n* *= 388*	Females *n* *= 267*	Males *n* *= 121*
Completion of upper secondary school by ≤ 21 years of age	**0.77** (0.76–0.78)	**0.81** (0.79–0.82)	**0.74** (0.72–0.75)	**0.63** (0.58–0.68)	**0.65** (0.59–0.71)	**0.63** (0.54–0.72)
Completion of upper secondary school by ≤ 25 years of age	**0.85** (0.84–0.86)	**0.87** (0.86–0.88)	**0.83** (0.81–0.84)	**0.75** (0.70–0.79)	**0.76** (0.71–0.81)	**0.75** (0.67–0.83)
Entered higher education by ≤ 25 years of age (completers of academic program^2^)	**0.92 ** (0.91-0.93)	**0.94 ** (0.93-0.95)	**0.88** (0.86-0.90)	**0.86 ** (0.80-0.91)	**0.89 ** (0.83-0.95)	**0.82 ** (0.70-0.93)

^1^ Adjusted predictions

^2^ in upper secondary school

Analyses are adjusted for sex, age, and family’s financial status

^*^Anxiety Disorders Interview Schedule for Children

## Discussion

This longitudinal, population-based study including more than 8,000 Norwegian adolescents showed a lower completion rate of upper secondary school among adolescents with social anxiety symptoms compared to those with few or no symptoms. Among those who completed an academic program in upper secondary school, the vast majority entered higher education by 25 years of age, although the tendency was somewhat lower among those with elevated symptom levels. Overall, our results are in accordance with existing evidence, mainly among individuals diagnosed with SAD [18–20, 22], and contribute to new knowledge by inclusion of both self-reported (questionnaire) and diagnostic screening information (interview) of social anxiety, and registry based educational data in a longitudinal study among community dwelling individuals.

### General discussion

The results of our study are in agreement with the vast majority of previous research linking SAD to low educational attainment [18–20]. To our best knowledge, only one previous study has used registry-based educational data investigating this relationship. Namely, the comprehensive study by VilaPlana-Pérez and authors [20] that reported lower odds for upper secondary school completion among individuals with a diagnosis of SAD according to the Swedish National Patient Registers. The results from our study indicate that the lower tendency to complete upper secondary school could be found also among socially anxious adolescents who do not necessarily fill all the diagnostic criteria for a diagnosis of SAD, and who have not necessarily sought professional help for their problems. In contrast to our findings, a Finish study investigating school dropouts prospectively among 15-yearolds did not find an association between self-reported SAD and dropouts at age 17 [51]. Additionally, a cross-sectional study investigating associations between early-onset mental disorders and educational attainment in a community sample of South African adults did not find a significant association between a lifetime diagnosis of SAD and non-completion of upper secondary school [52]. It is possible that the discrepancies to our study could be explained by the age of investigation and/or the operationalization of school dropouts. In the Finish study, dropouts were evaluated at a considerably lower age than in our study and defined as not being in school or work. This may hamper direct comparison of results with our study. The South African study included participants diagnosed with SAD aged 18 and older, and the information on completion of upper secondary school was based on recall. And, since the manifestation of social anxiety is shown to be culturally dependent [53], it is likely that cultural differences could also contribute to explain the discrepancies between our results and the results of the South African study. According to recent OECD-reports [54], 15% of youths aged 15–19 years in South Africa, are not registered in employment, education and/or training, compared to 3% in Norway.

There are several possible mechanisms that might contribute to lower completion rates of upper secondary school among adolescents with social anxiety. For instance, educational activities require engaging in various social and performative situations (e.g., public speaking, talking to authority figures, collaborating with classmates, and socializing with pupils outside the classroom) that might be distressing for individuals with social anxiety [12–14, 28, 55]. One suggested explanation is that experiencing anxiety in a school situation generates difficulties with concentrating in class, which in turn, affect academic performance [56]. Since one of the main characteristics of social anxiety is the avoidance of anxiety-triggering situations, it has also been suggested that experiencing fears might lead to school refusal and absence [57, 58], which in turn, could lead to dropouts.

The proportion of the completers of an academic program that was enrolled in higher education was lower in the SP group than in the SN group. Also, the predicted probability for enrollment was lower for the completers of academic programs in the SP group than for completers of academic programs in the SN group. This is in line with previous reports of a lower tendency among individuals with SAD to start a university degree [20, 22]. Since most students have to move away from their homes and families in order to live close to campus, involving establishment of new social networks, this process might be perceived as especially troublesome for adolescents with social anxiety [22]. The predicted probabilities for enrollment in higher education were high for academic completers in both SP and SN groups (86% and 92%), and for all levels of self-reported social anxiety symptoms (Fig. 2). Therefore, our results indicate that adolescents with social anxiety have greater impairment in upper secondary school completion than in start of higher education. It is important to note however, that socially anxious adolescents having completed upper secondary school most likely represent a selected group, affecting the results of higher education. Furthermore, our results provide information on *enrollment* in higher education, and not study completion. Previous research on university students suggests that social anxiety has negative impact on learning activities, well-being and academic performance [59, 60]. Further studies should therefore use longer observation periods of registry data in order to investigate study completion among young adults with social anxiety.

### Strengths and limitations

Important strengths of this study are the high participation rate (78.4%), its longitudinal design (i.e., each participant was followed until the age of 25 years), and the inclusion of social anxiety as both the participant’s ADIS-C screening status (interview) and the information that they self-reported relating to their social anxiety symptoms (questionnaire). Furthermore, as the information on completion of upper secondary school and enrollment in higher education was based on national registry data, we eliminated the risk of recall bias. Additionally, the use of a population sample when studying social anxiety is favorable, as it includes both already diagnosed cases and those who have not sought professional help for their problems. We also conducted sensitivity analyses that indicate that symptoms of social anxiety both at 13–15 years of age, and 16–19 years of age play a role in school completion.

Regarding limitations, it is expected that both mental health problems and the non-completion of upper secondary school may have been more common among non-responders in this school-based survey [32]. If this is the case, the negative association between social anxiety and education may have been underestimated in our study. The upper secondary school completion rates in official statistics fluctuate between 78% and 83% approximately, after 25 years of age [61]. In our dataset, using our population-based sample, estimates were 78–84% and indicate a representative sample.

In our statistical analyses, we chose to only adjust for sex, age, and socioeconomic status (SES). Notably, whether or not an individual completes upper secondary school and starts higher education is a complex interaction involving a variety of individual and contextual factors [62], such as family background and parental education [63, 64], academic performance and school motivation [65, 66], health related factors [67], as well as factors related to the school structure (62, 64). Also notably, our study used a subjective measure of socioeconomic status, as our data did not contain objective measures such as parental education or income. There is a possibility that use of a subjective measure of SES could be affected by some of the associated symptomatology of social anxiety, namely the tendency of increased social comparison, sense of inferiority and the tendency of self-devaluation [68]. However, since familial background has been shown to influence both mental health problems [69] and educational attainment [4], we found it necessary to include this. Finally, it is important to be aware that comorbid mental health problems are common among adolescents with SAD [17, 34, 70] and likely to contribute to the negative association with school completion. In a population-based Norwegian study investigating the impact of adolescent mental health problems on educational attainment, the negative association between internalizing symptoms and years of education, diminished when controlling for externalizing problems [71].

## Conclusion

Our findings show that adolescents with social anxiety have lower completion rates of upper secondary school at 21 and 25 years of age, and to some extent lower enrollment into higher education. Social anxiety symptoms typically emerge early in adolescence and are associated with low rates of help-seeking, and our results are of relevance to teachers, health and school professionals, as well as policy makers. Within the educational system, knowledge of typical symptoms and behaviours in order to identify pupils at risk, as well as universal health promotive and indicative preventive measures is of importance. As is a good collaboration between the school and the healthcare system in order to tailor tuition and professional help for pupils at risk.

### Electronic supplementary material

Below is the link to the electronic supplementary material.

Supplementary Material 1

## Data Availability

The Trøndelag Health Study (HUNT) has invited persons aged 13–100 year to four surveys between 1984 and 2019. Comprehensive data from more than 140,000 persons having participated at least once and biological material from 78,000 persons are collected. The data are stored in HUNT databank and biological material in HUNT biobank. HUNT Research Center has permission from the Norwegian Data Inspectorate to store and handle these data. The key identification in the data base is the personal identification number given to all Norwegians at birth or immigration, whilst de-identified data are sent to researchers upon approval of a research protocol by the Regional Ethical Committee and HUNT Research Center. To protect participants’ privacy, HUNT Research Center aims to limit storage of data outside HUNT databank, and cannot deposit data in open repositories. HUNT databank has precise information on all data exported to different projects and are able to reproduce these on request. There are no restrictions regarding data export given approval of applications to HUNT Research Center. For more information see: http://www.ntnu.edu/hunt/data. Requests to access the datasets should be directed to hunt.db@medisin.ntnu.no. The corresponding author could also be contacted with questions regarding data availability. Requests to access registry data from Statistics Norway should be directed to mikrodata@ssb.no.

## Abbreviations

SAD: Social Anxiety Disorder
Young-HUNT: Adolescent part of the Nord-Trøndelag Health Study (HUNT)
ADIS-C: Anxiety Disorders Interview Schedule for Children
ADIS-C SP: Anxiety Disorders Interview Schedule for Children Screening Positive
ADIS-C SN: Anxiety Disorders Interview Schedule for Children Screening Negative
NUDB: Norwegian National Education Database
DSM-IV: Diagnostic and Statistical Manual 4th Edition
SPAI-C: Social Phobia and Anxiety Inventory for Children
SPAI: Social Phobia and Anxiety Inventory
