# Supplementary material for: School completion and progression to higher education in adolescents with social anxiety: a linkage between Young-HUNT3 and national educational data (2008–2019), Norway
Source: BMC Public Health. 2024 Mar 18;24:833. doi: 10.1186/s12889-024-18271-w (PMC10946117; doi:10.1186/s12889-024-18271-w)
Supplement: Supplementary file 1 — Supplementary Material 1 [file 12889_2024_18271_MOESM1_ESM.docx]

**Figure S1.** Predicted probability and 95% confidence intervals for the completion of upper secondary school by ≤ 21 and ≤ 25 years of age according to sex and social anxiety disorder symptom index values of 1–5, and frequency of social anxiety disorder symptom index^1^ (bars), for adolescents aged 13-15 years.

^1^ The SAD symptom index is a mean score based on six items assessing social anxiety symptoms in various social situations, each rated on the following Likert scale: 1 = *never*, 2 = *seldom*, 3 = *sometimes*, 4 = *often*, and 5 = *always*. Thus, a SAD symptom index score of 1 is equal to “never” for all six items, and a SAD index score of 5 is equal to “always” for all six items.

**Figure S2:** Predicted probability and 95% confidence intervals for the completion of upper secondary school by ≤ 21 and ≤ 25 years of age according to sex and social anxiety disorder symptom index values of 1–5, and frequency of social anxiety disorder symptom index^1^ (bars), for adolescents aged ≥ 16 years.

^1^ The SAD symptom index is a mean score based on six items assessing social anxiety symptoms in various social situations, each rated on the following Likert scale: 1 = *never*, 2 = *seldom*, 3 = *sometimes*, 4 = *often*, and 5 = *always*. Thus, a SAD symptom index score of 1 is equal to “never” for all six items, and a SAD index score of 5 is equal to “always” for all six items.
